# Supplementary figures and images for: Altered Regional Activity and Network Homogeneity within the Fronto-Limbic Network at Rest in Medicine-Free Obsessive–Compulsive Disorder
Source: Brain Sci. 2022 Jun 29;12(7):857. doi: 10.3390/brainsci12070857 (PMC9312952; doi:10.3390/brainsci12070857)

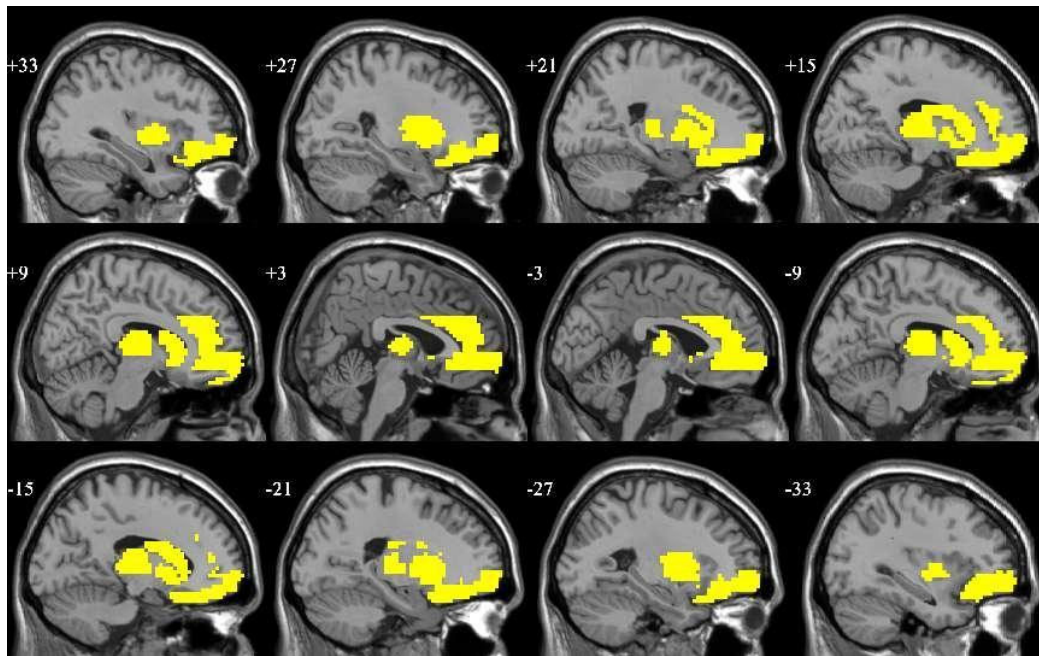

**Figure S1.** Fronto-limbic network mask.

Supplement: Supplementary file 1 [file brainsci-12-00857-s001.zip › brainsci-1759000-supplementary.pdf]
